# Draft Genome Sequence of Bradyrhizobium denitrificans K2, Cultivated from an Air Ventilation Environment

**DOI:** 10.1128/mra.00907-22

**Published:** 2022-11-08

**Authors:** Dhiraj Paul, Inga Paasisalo, Markku Katainen, Steffi Goffart, Jukka Pumpanen, Henri M. P. Siljanen

**Affiliations:** a Department of Environmental and Biological Sciences, University of Eastern Finland, Kuopio, Finland; University of Southern California

## Abstract

Bradyrhizobium denitrificans K2, isolated from an air circulation environment, has potential genes participating in inorganic nitrogen and carbon cycling. The draft genome comprises 8.31 Mb, with 7,982 coding sequences and 64.81% average G+C content. Genes related to carbon and inorganic nitrogen cycling were observed in the draft genome.

## ANNOUNCEMENT

The atmosphere is partly a living organism because of microbial compartments in the organic matter particles in the atmospheric particles, and this community is changing over the seasons due to atmospheric deposition (1). Air ventilation devices collect the air into the filters before entering into the housing systems. In the present study, we have isolated and performed the genome sequencing of the *Bradyrhizobium* sp., isolated from an air ventilation device. The genetic potential of this organism to participate in nitrogen and carbon cycling and its capabilities of playing a role in biogeochemical cycles are identified.

The isolation was started from the in-flow air ventilation device of the University of Eastern Finland, Canthia building, Kuopio, Finland. The filter was a bag filter of F9 with a glass-wool membrane (Hi-Flo; Camfil, Sweden) membrane (98% of PM_10_ particles taken). The air velocity in the device was 7 m^3^ s^−1^, with a surface area of filters of 36.8 m^2^ (one filter, 6.1 m^2^ per filter), and sampling time was 30 days. A 10-cm^2^ section of the F9 filter was added to the diluted nutrient broth (DNB) (2). The enrichment was done with an anoxic headspace with N_2_ and 5,000 ppb of nitrous oxide (N_2_O) as electron acceptor. The enrichment was done with 24 days passage time until growth was reached at the plateau phase. The enrichment was serial diluted to 10^2^ to 10^6^, and the isolate was streaked on 1.5% solidified DNB agar in the anoxic growth chamber with N_2_ headspace and 5,000 ppb of N_2_O. A pure colony was taken and grown for 24 days in liquid media (DNB) containing N_2_O in the headspace. After growth, a 60-mL broth culture was taken, and centrifugation was done. The pellet was used for DNA extraction. DNA extraction was done using the cetyltrimethylammonium bromide (CTAB) method with phenol-chloroform-isoamyl alcohol extraction followed by small modifications (3). The taxonomic identification of the organism was confirmed by PCR of the 16S rRNA gene with primers 27F and 1392R followed by Sanger sequencing (4). The best hit was shown with Bradyrhizobium denitrificans (GenBank accession no. NH041827) with 99.7% similarly.

The quality and quantity of the extracted DNA were checked and measured on an agarose (0.8%) gel and Qubit double-stranded DNA (dsDNA) high-sensitivity (HS) assay kit, respectively. The DNA fragmentation and library construction were done using a Nextera DNA Flex library preparation kit (Illumina, USA). Sequencing was done using 2 × 150-bp paired-end chemistry in the Illumina HiSeq platform (Microsynth, Switzerland).

A total of 102,353,882 paired-end reads were obtained. Sequence quality was checked using FastQC v0.11.9 (5) and trimmed using Trimmomatic v0.39 (6). Good quality reads were used for *de novo* assembly using SPAdes v3.15.4 (7). The assembly quality was checked by using QUAST v5.0.5 (8). Protein-encoding genes, tRNA genes, and rRNA genes were predicted using Prodigal (9), tRNA_scan-SE (10), and RNAmmer (11), respectively. Default parameters were used for all software. Genome assembly was annotated with the NCBI Prokaryotic Genome Annotation Pipeline (PGAP; https://www.ncbi.nlm.nih.gov/genome/annotation_prok/).

The draft genome sequence of Bradyrhizobium denitrificans K2 contains 8,283,947 bp with an average G+C content of 64.81%. All generated reads were assembled into 101 contigs (*N*_50_, 154,070 bp) with an estimated 1,700× coverage. The genome sequence included 7,440 candidate protein-coding genes (coding DNA sequences [CDSs]) and encoded at least 54 predicted RNAs, including 4 rRNAs and 50 tRNAs. The genome contained gene clusters for C_1_ to C_6_ carbon and inorganic nitrogen cycling, including denitrification in the environment.

### Data availability.

This whole-genome project has been deposited at DDBJ/EMBL/GenBank under the accession number JAMKCE000000000. The raw sequence is available at the NIH Sequence Read Archive (SRA) under the accession number SRP390381.
